# Interleukin-6 and C-reactive protein prognostic value following acute coronary syndrome

**DOI:** 10.1093/eurheartj/ehag317

**Published:** 2026-05-26

**Authors:** Johan Wouter Jukema, Nicolaas J Van Neer, Michael Szarek, Christa Cobbaert, Vera A Bittner, Markus Schwertfeger, Deepak L Bhatt, Sergio Fazio, Shaun G Goodman, Robert A Harrington, Irena Stevanovic, Harvey D White, Philippe Gabriel Steg, Gregory G Schwartz

**Affiliations:** Department of Cardiology, Leiden University Medical Center, PO Box 9600, Leiden 2300 RC, The Netherlands; Netherlands Heart Institute, Postbus 19258, 3501 DG Utrecht, The Netherlands; Department of Cardiology, Leiden University Medical Center, PO Box 9600, Leiden 2300 RC, The Netherlands; School of Public Health, Suny Downstate Medical Center, Brooklyn, USA; Mount Sinai Fuster Heart Hospital, Icahn School of Medicine at Mount Sinai, New York, NY, USA; Division of Cardiology, University of Colorado School of Medicine, Aurora, CO, USA; CPC (Colorado Prevention Center) Clinical Research, Aurora, CO, USA; Department of Cardiology, Leiden University Medical Center, PO Box 9600, Leiden 2300 RC, The Netherlands; Division of Cardiovascular Disease, University of Alabama at Birmingham, Birmingham, AL, USA; Medical Affairs, Roche Diagnostics Int. Ltd, Rotkreuz, Switzerland; Mount Sinai Fuster Heart Hospital, Icahn School of Medicine at Mount Sinai, New York, NY, USA; Scientific Council, Cardiovascular and Metabolic Diseases, Regeneron Pharmaceuticals, Inc., Tarrytown, NY, USA; Division of Cardiology, Department of Medicine, Canadian VIGOUR Centre, University of Alberta, Edmonton, Alberta, Canada; Division of Cardiology, Department of Medicine, St Michael's Hospital, University of Toronto, Toronto, Ontario, Canada; Dean, Weill Cornell Medicine, New York, NY, USA; Dyslipidemia Medical Affairs, Sanofi, Paris, France; Green Lane Cardiovascular Research Unit, Te Whatu Ora—Health New Zealand, Te Toka Tumai, and University of Auckland, Auckland, New Zealand; Université Paris-Cité, UMR 1148/LVTS, FACT/F-CRIN (French Alliance for Cardiovascular Trials), AP-HP, Paris, France; Division of Cardiology, University of Colorado School of Medicine, Aurora, CO, USA

**Keywords:** Interleukin-6, Cardiovascular events, PCSK9, Cholesterol, Acute coronary syndrome, High-sensitivity C-reactive protein

## Abstract

**Background and Aims:**

After acute coronary syndrome (ACS), high-sensitivity C-reactive protein (hsCRP) and interleukin-6 (IL-6) levels have been associated with risk of major adverse cardiovascular events (MACE). Whether the prognostic information provided by hsCRP and IL-6 is independent and complementary after ACS is unclear.

**Methods:**

The ODYSSEY OUTCOMES trial compared alirocumab with placebo in post-ACS patients on optimized statin therapy. In *post hoc* analyses, the relation between log-transformed hsCRP and IL-6 levels and risk of MACE and all-cause death was assessed in proportional hazards models.

**Results:**

A total of 11 817 patients had baseline hsCRP and IL-6 data; 1306 had a MACE primary endpoint and 458 died. Median hsCRP, IL-6, and low-density lipoprotein cholesterol (LDL-C) were 1.54 mg/L, 5.00 pg/ml, and 86 mg/dl, respectively. hsCRP and IL-6 had moderate correlation (*r* = 0.52). In models for one biomarker adjusted for the other biomarker, treatment assignment, age, sex, diabetes, LDL-C, and time since index ACS, hsCRP was a significant independent predictor of MACE (*P* < .0001) and IL-6 was not (*P* = .21); both independently predicted death (*P* < .0001 for hsCRP and *P* = .0003 for IL-6). Relationships did not depend on treatment (all *P_interaction_* > .25). When dichotomized at 2 mg/L (hsCRP) and 5 pg/ml (IL-6), risks of MACE and death were significantly elevated when both, but not when one marker was elevated.

**Conclusions:**

In patients with recent ACS, hsCRP independently predicted MACE, while both hsCRP and IL-6 predicted death. Together, the two markers provided complementary prognostic information. hsCRP conveys independent information on inflammatory risk beyond that provided by IL-6.

## Structured Graphical Abstract

**Structured graphical abstract:**
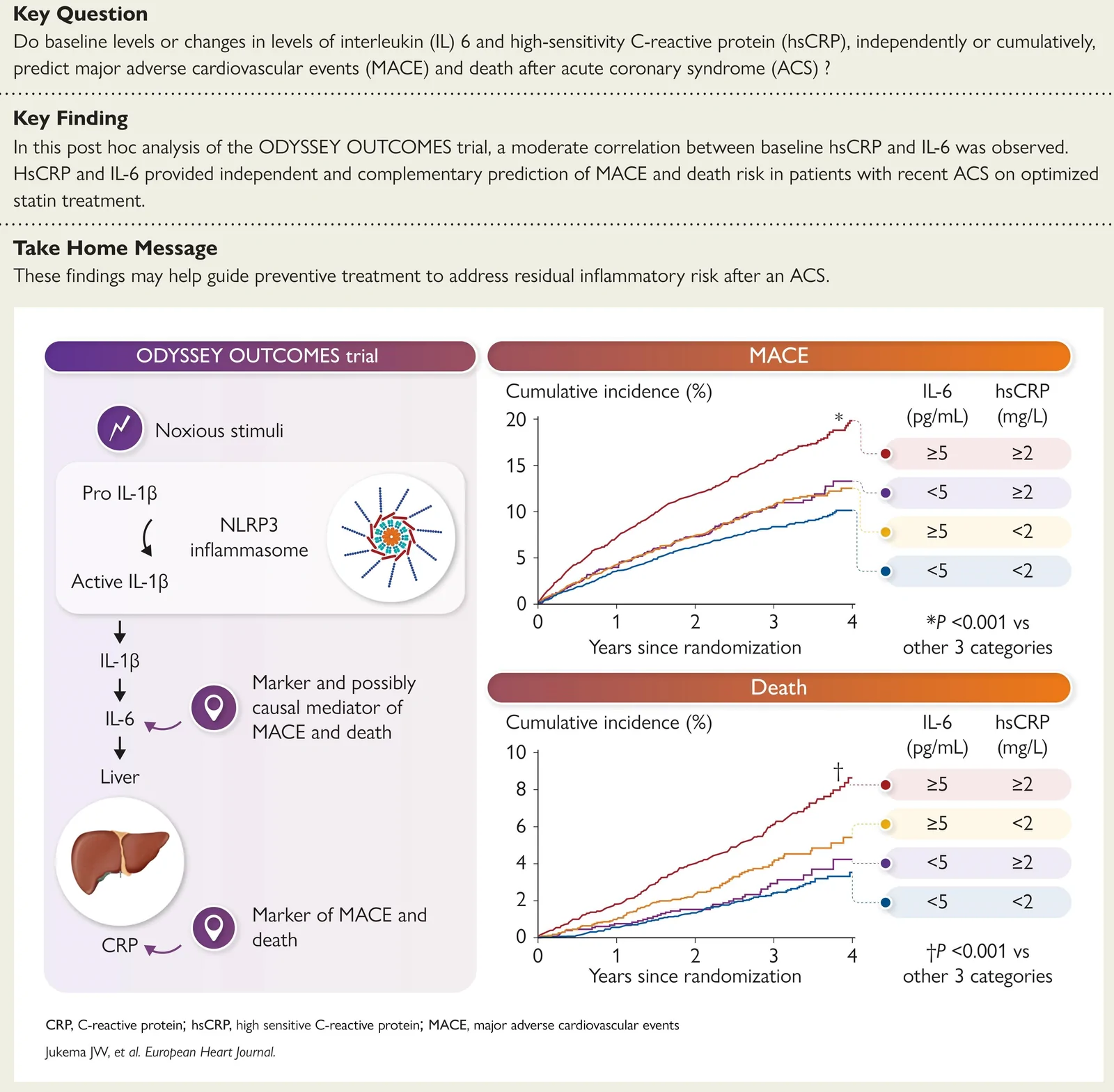
Inflammasomes are key signalling platforms that detect noxious stimuli and activate the highly pro-inflammatory cytokine, IL-1β, which stimulates elaboration of IL-6 by inflammatory cells and CRP by the liver. When dichotomized at conventional thresholds (5 pg/ml for IL-6; 2 mg/L for hsCRP), MACE and death following ACS were significantly higher only when both biomarkers were elevated. ACS, acute coronary syndrome; CI, confidence interval; CRP, C-reactive protein; hsCRP, high-sensitivity C-reactive protein; IL, interleukin; MACE, major adverse cardiovascular events; NLRP3, nucleotide-binding leucine-rich repeat-containing pyrine receptor 3.

**See the editorial comment for this article ‘Multi-marker inflammatory risk assessment after acute coronary syndrome’, by E.S.G. Stroes and M.E. Annink, https://doi.org/10.1093/eurheartj/ehag502.**

## Introduction

Systemic inflammation plays a central role in the pathophysiology of atherosclerosis and acute coronary syndrome (ACS).^1^ Acute phase responses and immune reactions activate the nucleotide-binding leucine-rich repeat-containing pyrine receptor 3 (NLRP3) inflammasome, in turn causing activation of interleukin (IL)-1β and production of IL-6 by inflammatory cells. In the liver, IL-6 stimulates production of the widely used downstream clinical biomarker C-reactive protein (CRP). Activation of the immune system/inflammasome by noxious stimuli, including low-density lipoprotein cholesterol (LDL-C), may destabilize atherosclerotic plaques, thereby increasing vulnerability rupture and risk of major adverse cardiovascular events (MACE).^2,3^

An elevated level of either IL-6 or high-sensitivity CRP (hsCRP) is associated with poor cardiovascular outcomes and in the 2024 European Society of Cardiology guidelines on chronic coronary syndromes, there is increasing awareness of the importance of inflammation, and a recommendation to consider the determination of inflammatory markers for risk stratification and treatment decisions.^4–7^ Hepatic CRP expression is not, however, solely dependent on the level of IL-6 stimulation. Other factors can modify the expression of CRP by the liver. For example, statin treatment may reduce CRP expression by hepatocytes by reducing nuclear translocation of nuclear factor-κB or increasing nitric oxide release, independent of IL stimulation.^8,9^ Therefore, it is reasonable to hypothesize that upstream causal (IL-6) and downstream transduced (CRP) biomarkers may convey independent clinical information related to prognosis, particularly in statin-treated patients. However, data on whether baseline levels, or changes in the levels of these two biomarkers, independently or cumulatively predict MACE and death after ACS are lacking.

The ODYSSEY OUTCOMES trial, which evaluated the proprotein convertase subtilisin/kexin type 9 (PCSK9) inhibitor alirocumab in post-ACS patients on optimized statin therapy, provided an opportunity to address these questions in a high-risk, well-treated population.^10^

## Methods

This investigation is a prespecified substudy of the ODYSSEY OUTCOMES trial (NCT01663402), a Phase 3, multicentre, randomized, double-blind, placebo-controlled trial that evaluated the long-term cardiovascular effects of alirocumab, a monoclonal antibody targeting PCSK9, in patients who had experienced an ACS 1 to 12 months before randomization. All patients received optimized statin therapy, defined as high-intensity or the maximum tolerated dose.

Eligible participants were adults (≥40 years) who had been hospitalized with a recent ACS. For the biomarker analysis, blood samples were available from a predefined biobank subset of 11 817 patients at baseline and 10 933 patients at 4 months after randomization. Samples were stored at −80°C until batch analysis. Measurements of hsCRP and IL-6 were performed using high-sensitivity immunoturbidimetric and electrochemiluminescence immunoassays, respectively (Roche Diagnostics, Zug, Switzerland). Levels of detection of these intensively validated essays were 0.150 mg/L for hsCRP and 1.5 pg/ml for IL-6, respectively.

The primary endpoint was MACE, a composite of coronary heart disease death, non-fatal myocardial infarction, fatal or non-fatal ischaemic stroke, or unstable angina requiring hospitalization. All-cause death was a secondary endpoint. Outcomes were adjudicated by physicians blinded to treatment assignment.

The trial adhered to Good Clinical Practice and the Declaration of Helsinki. Informed consent was obtained from all participants. The trial was funded by Sanofi and Regeneron Pharmaceuticals. Analysis of biomarkers was supported by Roche Diagnostics.

### Statistical analysis

Analyses were performed on data from all randomized participants who had available baseline hsCRP and IL-6 assessments. Categorical variables are summarized by frequency and percentage of participants, while continuous variables are summarized by median and interquartile range. Comparisons of change from baseline to month 4 in hsCRP, IL-6, and LDL-C were done by Wilcoxon tests.

Owing to right-skewed distributions, log-transformed hsCRP and IL-6 values were related to time-to-event outcomes (figures reflect back-transformation). The continuous relationships between baseline values of each biomarker and cumulative incidence of MACE or death 4 years after randomization were estimated by restricted cubic splines from proportional hazards models with knots at the 25th, 50th, and 75th percentiles of the biomarker. The relationships for each biomarker reflected adjustment for a spline of the other biomarker, treatment assignment, age, sex, diabetes, baseline LDL-C, and time from index ACS to randomization. Similar analyses were performed for the change in each biomarker from baseline to month 4 and subsequent events. In addition to the adjustment factors from the baseline models, the change models for each biomarker included adjustment for a spline of change in the other biomarker and change in LDL-C. Hazard ratios (HR) were also expressed in terms of a one standard deviation increase in the change in each biomarker. Potential interactions between each of the splines and treatment assignment were assessed by adding interaction terms to the models.

Cumulative incidence of MACE or death by baseline biomarker categories dichotomized at conventional risk thresholds—2 mg/L for hsCRP, 5 ng/L for IL-6—were estimated by Kaplan–Meier methods. Comparisons between the four categories were made via proportional hazards models with adjustment for treatment assignment, age, sex, diabetes, baseline LDL-C, and time from index ACS to randomization.

To provide further context for the independent prognostic information provided by hsCRP and IL-6, a concordance analysis was performed to determine whether consideration of each biomarker individually or both jointly improves the concordance of observed vs predicted outcomes among all possible pairs of participants in which one participant has a MACE or dies and the other participant does not.

## Results

Patients were enrolled a median of 2.7 months after ACS. Clinical and laboratory characteristics of the analysis cohort at baseline are presented in Supplementary data online, *Appendix Table S1*. Patient characteristics and markers of inflammation at baseline stratified by baseline IL-6 and hsCRP are shown in *Table 1*. There were no significant differences between the alirocumab and placebo groups. *Figure 1* shows the distribution of baseline values for hsCRP and IL-6 in aggregate for both treatment groups. Baseline median (interquartile range) hsCRP was 1.54 (0.74, 3.56) mg/l and IL-6 was 5.00 (3.18, 6.89) pg/ml. Baseline hsCRP and IL-6 levels were moderately correlated (Spearman *r* = 0.52). During follow-up, 11.1% of the participants (*n* = 1306) experienced at least one MACE and 3.9% (*n* = 458) died. Overall, alirocumab reduced MACE (HR 0.82, 95% confidence interval [CI] 0.74–0.92; *P* = .0004) and death (HR 0.83, 95% CI 0.69–1.00; *P* = .04) in the analysis cohort.

**Figure 1:**
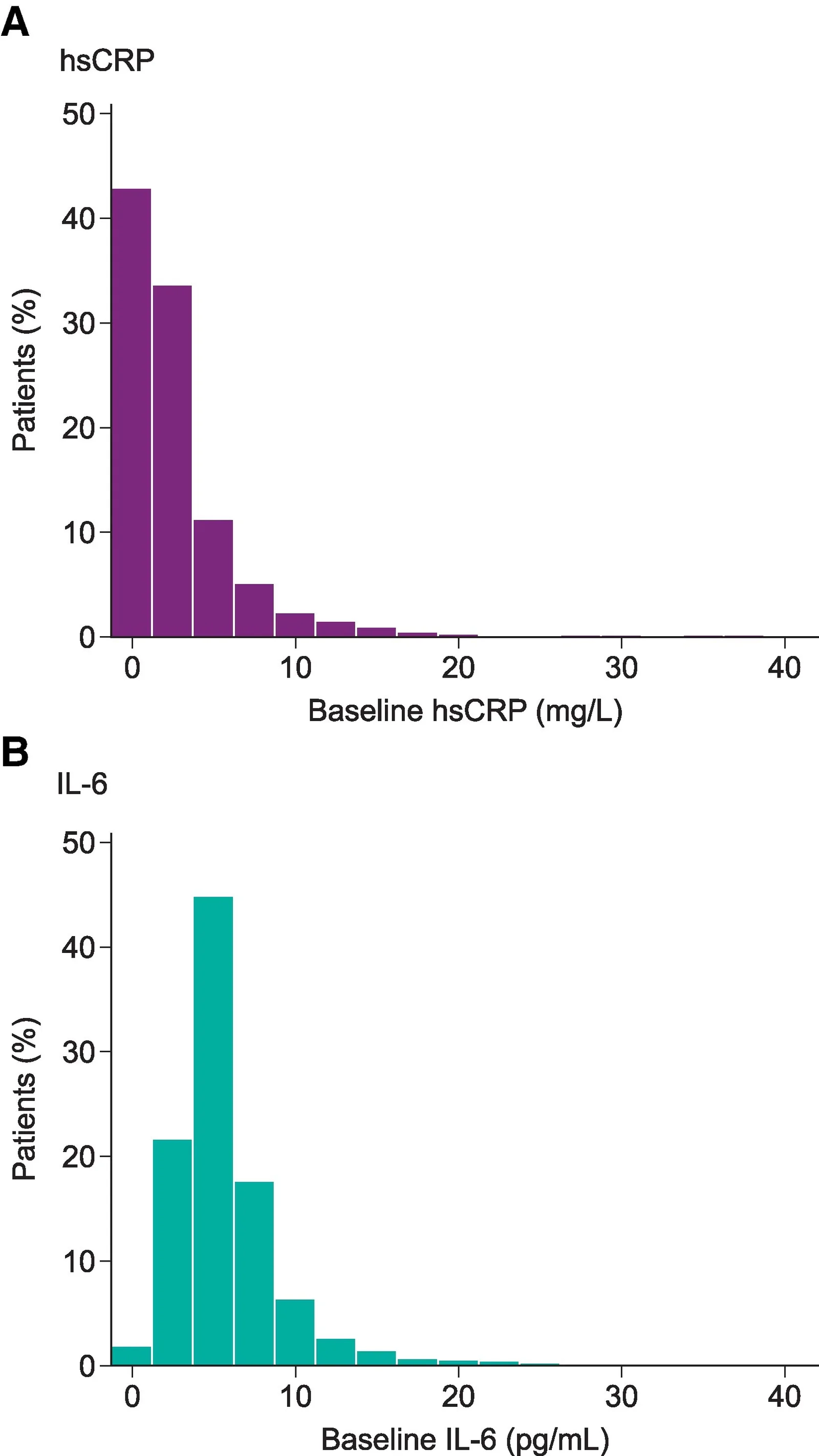
Baseline histograms for (*A*) hsCRP and (*B*) IL-6. hsCRP, high-sensitivity C-reactive protein; IL-6, interleukin-6

**Table 1:** Patient characteristics and markers of inflammation at baseline stratified by baseline IL-6 and hsCRP

Variable	IL-6 < 5 pg/ml, hsCRP <2 mg/l (*n* = 4587)	IL-6 < 5 pg/ml, CRP ≥2 mg/l (*n* = 1309)	IL-6 ≥ 5 pg/ml, hsCRP <2 mg/l (*n* = 2313)	IL-6 ≥ 5 pg/ml, CRP ≥2 mg/l (*n* = 3608)
Age, years	57 (51, 64)	55 (50, 62)	60 (53, 67)	59 (52, 66)
Female sex	898 (19.6)	325 (24.8)	542 (23.4)	1066 (29.5)
Region of **enrolment**				
Central and Eastern Europe	924 (20.1)	323 (24.7)	344 (14.9)	681 (18.9)
Western Europe	1226 (26.7)	334 (25.5)	495 (21.4)	744 (20.6)
Canada or USA	949 (20.7)	310 (23.7)	540 (23.3)	983 (27.2)
Asia	573 (12.5)	70 (5.3)	408 (17.6)	320 (8.9)
Rest of World	374 (8.2)	140 (10.7)	179 (7.7)	336 (9.3)
Hypertension	2645 (57.7)	803 (61.3)	1455 (62.9)	2552 (70.7)
HbA1c, %	5.7 (5.5, 6.1)	5.8 (5.6, 6.3)	5.9 (5.6, 6.4)	6.0 (5.6, 6.7)
eGFR, ml/min/1.73 m^2^	79.4 (69.8, 90.8)	82.3 (70.3, 95.8)	76.0 (64.3, 87.6)	77.0 (64.4, 89.7)
Diabetes	1017 (22.2)	361 (27.6)	742 (31.1)	1366 (37.9)
Current smoking	981 (21.4)	449 (34.3)	476 (20.6)	943 (26.1)
High-intensity statin treatment at **randomization**	4110 (89.6)	1153 (88.1)	2075 (89.7)	3181 (88.2)
Index acute coronary syndrome	*n* = 4580	*n* = 1307	*n* = 2310	*n* = 3602
Non-ST-segment elevation myocardial infarction	2176 (47.5)	640 (49.0)	1045 (45.2)	1793 (49.8)
ST-segment elevation myocardial infarction	1664 (36.3)	497 (38.0)	830 (35.9)	1263 (35.1)
Unstable angina	740 (16.2)	170 (13.0)	435 (18.8)	546 (15.2)
Time from index ACS to **randomization**, months	2.8 (1.8, 4.9)	2.8 (1.8, 5.0)	2.7 (1.7, 4.5)	2.5 (1.7, 4.0)
Revascularization for index ACS	3446 (75.1)	971 (74.2)	1692 (73.2)	2554 (70.8)
Baseline markers of inflammation				
hsCRP, mg/l	0.75 (0.47, 1.15)	3.15 (2.44, 4.43)	1.05 (0.65, 1.46)	4.76 (3.11, 7.99)
IL-6, pg/ml	3.73 (3.03, 4.30)	4.09 (3.40, 4.59)	6.17 (5.49, 7.42)	7.55 (6.15, 10.06)
Baseline lipids and lipoproteins				
LDL-C, mg/dl	86 (73, 102)	92 (77, 114)	83 (71, 100)	88 (73, 108)
Triglycerides, mg/dl	124 (89, 175)	141 (98, 201)	127 (94, 181)	136 (101, 188)
HDL-C, mg/dl	44 (38, 52)	42 (37, 50)	42 (36, 49)	40 (34, 47)
Lipoprotein(a), nmol/l	43.5 (12.7, 157.8)	39.1 (12.8, 152.1)	48.3 (13.1, 154.5)	46.6 (14.2, 149.5)

Numbers are *n* (%) or median (inter-quartile range).

hsCRP, high-sensitivity C-reactive protein; HDL-C, high-density lipoprotein cholesterol; IL-6, interleukin-6; LDL-C, low-density lipoprotein cholesterol.

*Figure 2* displays spline relations between continuous baseline hsCRP or IL-6 and MACE or death, adjusted for the other biomarker and baseline characteristics. Baseline hsCRP predicted MACE (spline *P* < .0001) but baseline IL-6 did not (spline *P* = .21), although the latter relationship remained directionally concordant. Both baseline hsCRP and IL-6, adjusted for each other and baseline characteristics, predicted death (spline *P* < .0001 and *P* = .0003, respectively). These relationships did not depend on treatment assignment (all spline × treatment *P*_interaction_ > .25).

**Figure 2:**
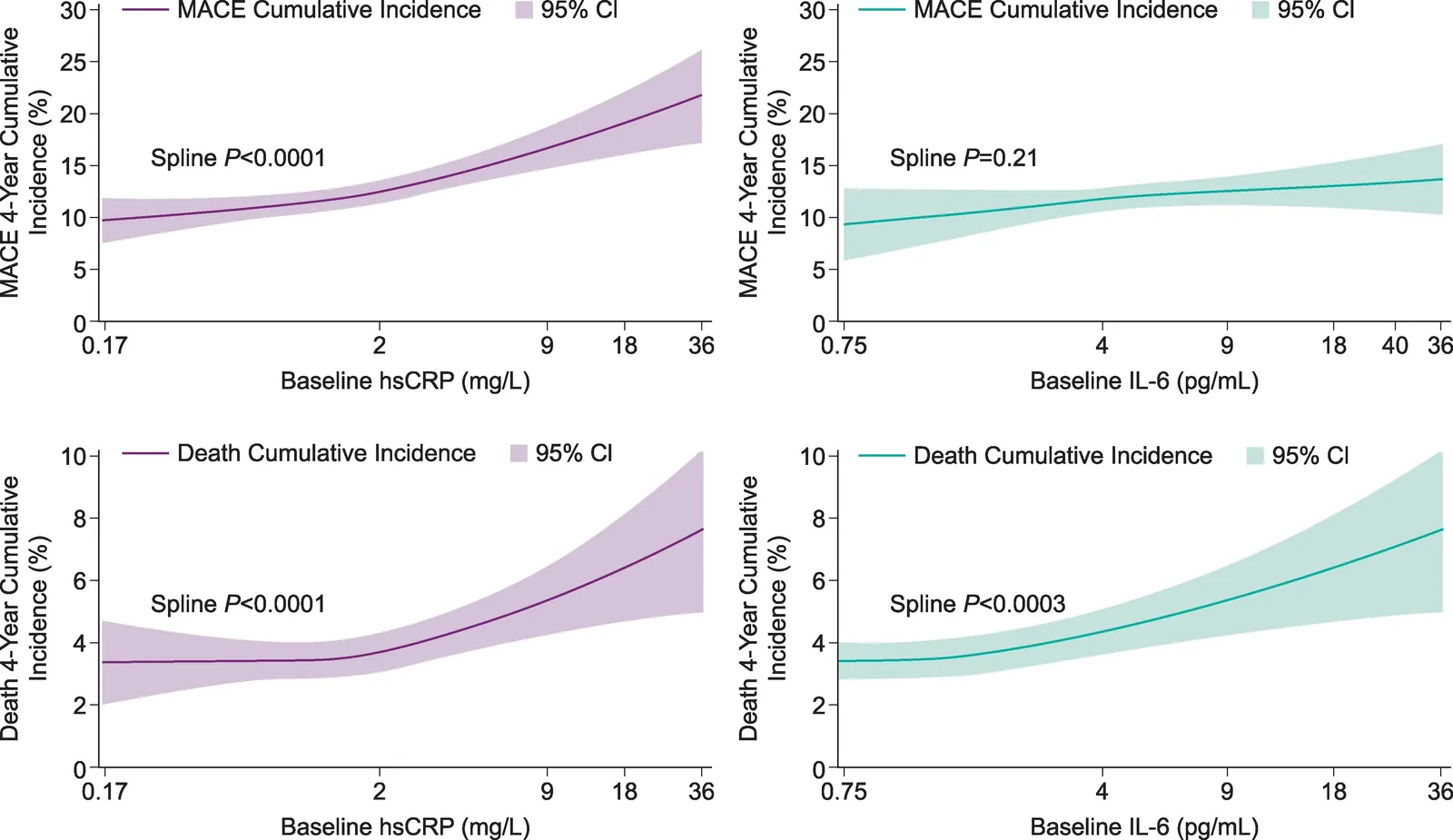
Restricted cubic splines for baseline log(hsCRP) and log(IL-6), adjusted for one another and for treatment assignment, age, sex, diabetes, baseline low-density lipoprotein cholesterol, and time to index acute coronary syndrome, with regard to cumulative incidence of MACE (top row) and death (bottom row) through 4 years after randomization. Knots are at the 25th, 50th, and 75th percentiles and span the approximate 1st to 99th percentiles of each biomarker and are estimated from proportional hazards models. Solid line indicates estimated 4-year cumulative incidence and shaded area indicates 95% CI. CI, confidence interval; hsCRP, high-sensitivity C-reactive protein; IL-6, interleukin-6; MACE, major adverse cardiovascular events

*Figure 3A* shows Kaplan–Meier plots for MACE stratified by baseline hsCRP and IL-6 dichotomized at conventional thresholds of 2 mg/L and 5 pg/ml, respectively. *Figure 3B* shows the analogous relations for death. Curves for subgroups with one or both biomarkers elevated at or above the threshold were compared with the referent subgroup with neither biomarker elevated to the threshold. For MACE, after adjustment for baseline characteristics, elevation of just one biomarker was not significantly associated with increased risk (elevated hsCRP, but not IL-6: HR 1.20; 95% CI 0.98–1.46; *P* = .07; elevated IL-6, but not hsCRP: HR 1.12; 95% CI 0.95–1.32; *P* = .17). However, when both biomarkers were elevated, there was significant and pronounced elevation in risk (HR 1.69, 95% CI 1.48–1.93; *P* < .0001).

**Figure 3:**
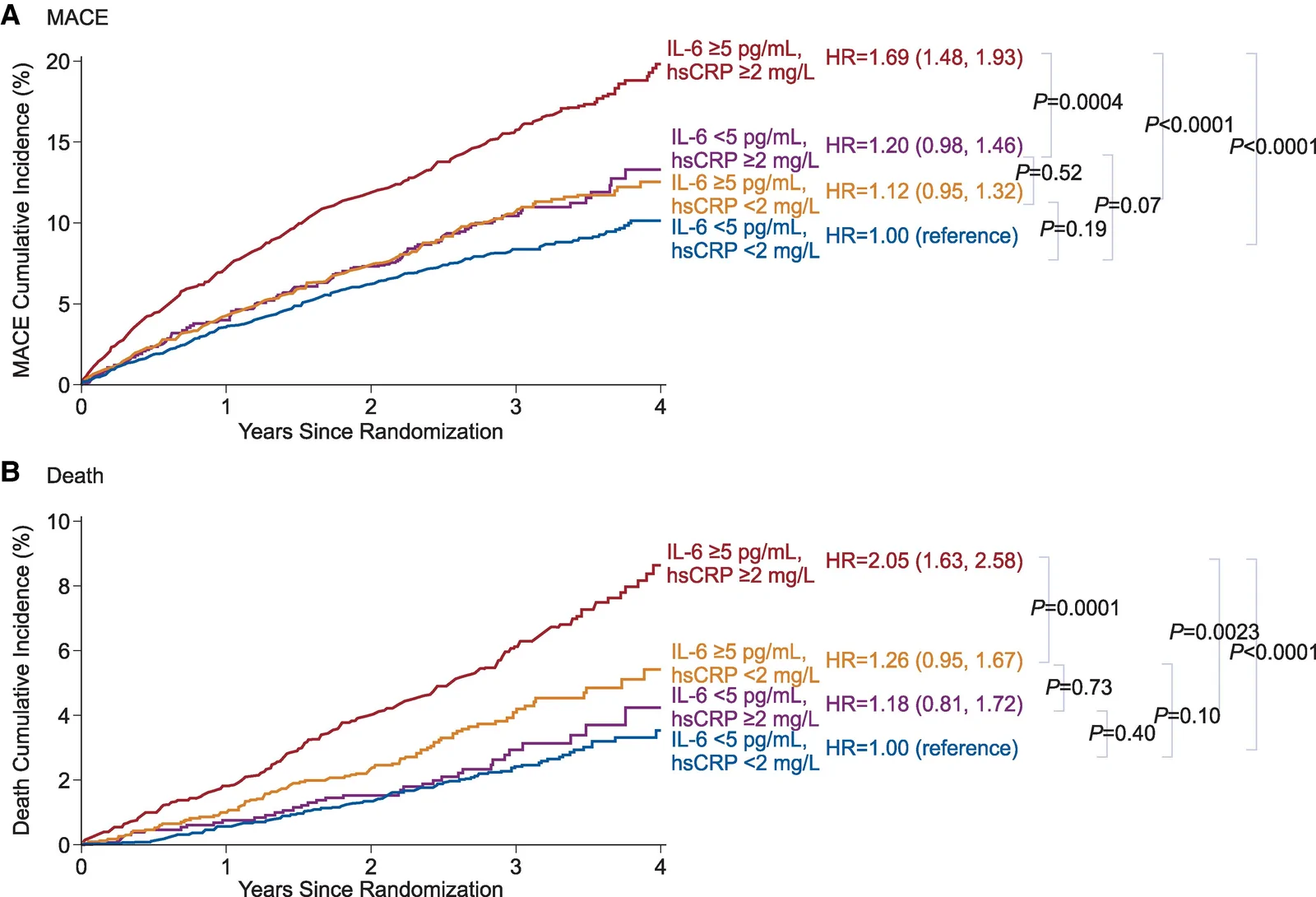
Kaplan–Meier cumulative incidence curves for (*A*) MACE and (*B*) death according to baseline hsCRP and IL-6 levels. Hazard ratios and *P*-values reflect adjustment for treatment assignment, age, sex, diabetes, baseline LDL-C, and time from index ACS to randomization. One elevated biomarker (based on a dichotomous criterion IL-6 ≥5 and hsCRP ≥2) increases risk. Both elevated biomarkers increase the risk significantly more. These two biomarkers together provide complementary prediction of elevated risk HR, hazard ratio; hsCRP, high-sensitivity C-reactive protein; IL-6, interleukin-6

For death, findings were analogous. After adjustment for baseline characteristics, one elevated biomarker was not significantly associated with increased risk (elevated hsCRP, but not IL-6: HR 1.18; 95% CI 0.81–1.72; *P* = .40; elevated IL-6, but not hsCRP: HR 1.26; 95% CI 0.95–1.67; *P* = .10). However, when both hsCRP and IL-6 were elevated above thresholds, the risk of death was markedly and significantly elevated (HR 2.05; 95% CI 1.63–2.58; *P* < .0001). Thus, jointly elevated hsCRP and IL-6 predicted the risk of MACE and death after ACS when either singly elevated biomarker did not.

Concordance analysis of pairs of participants whose observed and predicted MACE or death outcomes agree (were concordant) showed that for both outcomes CRP and IL-6 together significantly increased prediction more than either one individually (Supplementary data online, *Appendix Table S2*).

Supplementary data online, *Appendix Table S1* displays the changes in hsCRP, IL-6, and LDL-C from baseline to month 4 of assigned treatment. Changes in hsCRP and IL-6 were minimal in both treatment groups, indicating that the placebo group was at steady state after the index ACS. Although there were no systematic changes in the inflammatory markers in either treatment group over 4 months, there was substantial inter-individual variability in those changes as manifest by the broad interquartile ranges for the changes in Supplementary data online, *Appendix Table S1*. As expected, alirocumab produced a large reduction in LDL-C compared with placebo (*P* < .0001).

To determine whether inter-individual differences in changes in biomarker levels from baseline to month 4 reflected random variation or were clinically relevant, spline relations between the change in hsCRP or IL-6 from baseline to month 4 and the risk of MACE and death after month 4 were constructed. For both biomarkers and both clinical outcomes, a rise in biomarker concentration was associated with significantly greater clinical risk (*Figure 4*); similar to baseline relationships, these change relationships did not depend on treatment assignment (all spline × treatment *P_interaction_* > .25). Furthermore, a one standard deviation increase in change in each biomarker was associated with an approximate 10%–20% increased risk of MACE or death with no interaction with treatment (Supplementary data online, *Appendix Table S3*).

**Figure 4:**
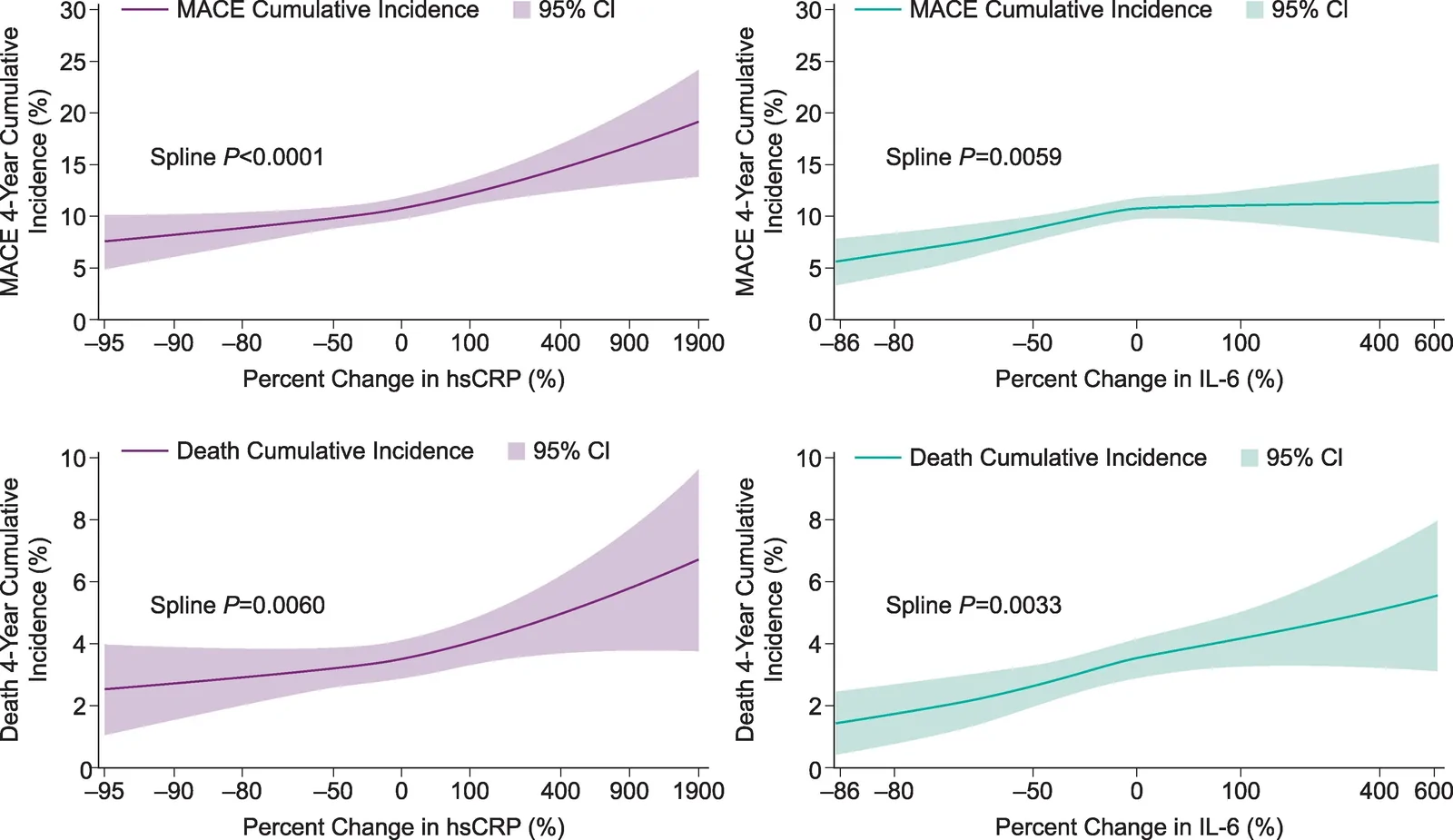
Spline curves with regard to change from baseline to Month 4 in log(hsCRP) and log(IL-6), adjusted for one another and for treatment assignment, age, sex, diabetes, baseline log(hsCRP), baseline log(IL-6), baseline low-density lipoprotein cholesterol, change in low-density lipoprotein cholesterol and time to index acute coronary syndrome, with regard to cumulative incidence of MACE (top panel) and death (bottom panel) through 4 years after randomization. Knots are at the 25th, 50th, and 75th percentiles and span the approximate 1st to 99th percentiles of each biomarker and are estimated from proportional hazards models. The solid line indicates the estimated 4-year cumulative incidence and the shaded area indicates 95% CI. CI, confidence interval; hsCRP, high-sensitivity C-reactive protein; IL-6, interleukin-6; MACE, major adverse cardiovascular events

## Discussion

There are several key findings from this analysis of a large cohort of patients with recent ACS receiving high-intensity or maximum tolerated statin therapy (*Structured Graphical Abstract*). First, a moderate correlation between baseline hsCRP and IL-6 was expected and was confirmed, consistent with their common biological pathways, with IL-6 being a proximal mediator of the hepatic acute-phase response leading to CRP expression.^4^

Second, despite the correlation between the two biomarkers, continuous hsCRP remained a robust independent predictor of MACE and death after adjustment for IL-6. As an inexpensive and widely available biomarker, the findings support the clinical utility of hsCRP in patients with recent ACS, notwithstanding most evidence against causal participation of CRP in atherothrombosis.^11,12^ Moreover, the finding that continuous hsCRP adjusted for IL-6 remained an independent predictor of MACE supports the concept of hsCRP as an integrative marker of systemic inflammation that may better reflect cumulative inflammatory risk than the more upstream IL-6.^2,4^ Nonetheless, IL-6 was associated with all-cause death independent of hsCRP and synergistically predicted MACE with hsCRP, supporting the relevance of IL-6 as a potential therapeutic target. As a central cytokine in the inflammatory cascade, elevated IL-6 levels may signal a chronic proinflammatory state not fully captured by downstream markers like hsCRP alone.^3^ Preclinical evidence points to IL-6 as a direct participant in atherothrombosis^13^ and Mendelian randomization analyses suggest that, in contrast to hsCRP, IL-6 is causal for atherosclerotic cardiovascular events.^13–15^

Third, strong, synergistic prognostic information for both MACE and death was provided when hsCRP and IL-6 were simultaneously elevated. Patients with elevations of both biomarkers at or above conventional thresholds had an estimated 69% higher risk of MACE and a 105% greater risk of death over a median follow-up of 2.6 years, compared to patients with neither biomarker elevated above those thresholds. Although IL-6 is seldom measured in clinical practice and is not incorporated in standard risk prediction models for patients with coronary heart disease, the present findings suggest that its incorporation along with hsCRP in such models may meaningfully improve the prediction of risk after ACS.

Fourth, despite substantial reductions in LDL-C beyond those attained with statin treatment alone, alirocumab had no effect on hsCRP or IL-6 levels. Nonetheless, alirocumab reduced the incidence of MACE and death.^6,10^ These observations align with previous work indicating that lipoprotein-attributable and inflammation-related risks are at least partly independent and that therapies targeting each risk factor are complementary.^1,11,12^ The absence of a systematic effect of alirocumab on hsCRP or IL-6 levels in the current analysis aligns with previous findings that PCSK9 monoclonal antibodies do not affect these biomarkers in patients with cardiovascular disease.^16^ However, our findings stand in contrast to those of Navarese *et al.* who showed that PCSK9 inhibition compared with placebo reduced IL-6 levels along with the risk of death or intubation in patients with severe COVID-19 who were specifically selected for very high baseline levels of IL-6, approximately 10-fold higher than in the current analysis cohort.^17^ Thus, it is possible that PCSK9 inhibitors may blunt the expression of IL-6 when the pathway is highly activated.

Fifth, though there was no overall change in either hsCRP or IL-6 levels between two post-ACS time points 4 months apart, there was substantial inter-individual variability in these measures. Patients who experienced a rise in hsCRP over this interval were at elevated risk of subsequent MACE and death, while the converse was true for patients with declining levels. For IL-6, patients who experienced a rise in levels had a greater subsequent risk of death, while those who experienced a fall in levels had a lower subsequent risk of MACE and death. Thus, serial assessment of these inflammatory markers may help refine the prognosis of patients who appear clinically stable after ACS.

The findings of the current report underscore the overlap as well as the distinctions between hsCRP as a biomarker of risk and IL-6 as a likely causal mediator of risk and potential target of therapy. In a *post hoc* analysis of the CANTOS trial, lowering IL-6 levels through IL-1β inhibition with canakinumab was associated with a reduced the risk of MACE after acute myocardial infarction.^18^ The safety and efficacy of direct IL-6 inhibition in cardiovascular disease is currently under active investigation, using drugs including tocilizumab, an IL-6–receptor inhibitor,^19,20^ ziltivekimab, a fully human monoclonal antibody targeting the IL-6 ligand,^21^ and other novel anti-IL-6 molecules.^22^ If IL-6 interventions demonstrate a clinical benefit that depends on baseline levels of IL-6, measurement of that biomarker may become even more relevant to clinical practice.

### Strengths and limitations

Among the strengths of the present analysis is its prespecified design in a large, well-characterized subset of a prospective randomized international trial with blindly adjudicated endpoints. Another key strength is the serial assessment of inflammatory biomarkers outside of the immediate post-ACS period. Previous studies have demonstrated that both hsCRP and IL-6 are elevated transiently in the early period after ACS.^4,6^ Those elevations may reflect the severity of acute myocardial injury but may not define long-term inflammatory risk. In contrast, in the current analysis cohort, there were no systematic changes in biomarkers from initial assessment at a median 2.7 months from ACS and a subsequent measurement 4 months later, thus defining a stabilized post-ACS cohort in which acute phase effects on biomarkers had subsided. Thus, the current data may be particularly informative of long-term risk related to stable inflammatory biomarker elevation after ACS. Among possible limitations of the current analysis is selection bias since samples were available for biomarker analysis only at sites that participated in this substudy, comprising approximately 62% of all ODYSSEY OUTCOMES participants. Nonetheless, the large size of the cohort provided sample provided substantial power for statistical inference.

## Conclusion

HsCRP and IL-6 provide independent and complementary prediction of the risk of MACE and death in patients with recent ACS on optimized statin treatment.

## Supplementary Material

ehag317_Supplementary_Data

## Declarations

### Data Availability

Requests from qualified investigators for data from the ODYSSEY OUTCOMES trial will be considered by its Executive Steering Committee at odysseyoutcomesESC@gmail.com.

### Pre-registered Clinical Trial Number

ClinicalTrials.gov ID NCT01663402.

## References

[1] Gabay C, Kushner I. Acute-phase proteins and other systemic responses to inflammation. N Engl J Med 1999;340:448–54. 10.1056/NEJM199902113400607 9971870

[2] Jukema RA, Ahmed TAN, Tardif JC. Does low-density lipoprotein cholesterol induce inflammation? If so, does it matter? Current insights and future perspectives for novel therapies. BMC Med 2019;17:197. 10.1186/s12916-019-1433-3 PMC6824020 31672136

[3] Stark K, Massberg S. Interplay between inflammation and thrombosis in cardiovascular pathology. Nat Rev Cardiol 2021;18:666–82. 10.1038/s41569-021-00552-1 PMC8100938 33958774

[4] Ridker PM . From C-reactive protein to interleukin-6 to interleukin-1: moving upstream to identify novel targets for atheroprotection. Circ Res 2016;118:145–56. 10.1161/CIRCRESAHA.115.306656 PMC4793711 26837745

[5] Kaptoge S, Seshasai SR, Gao P, Freitag DF, Butterworth AS, Borglykke A, et al Inflammatory cytokines and risk of coronary heart disease: new prospective study and updated meta-analysis. Eur Heart J 2014;35:578–89. 10.1093/eurheartj/eht367 PMC3938862 24026779

[6] Steg PG, Szarek M, Jukema JW, Bhatt DL, Bittner VA, Diaz R, et al Relation of low-density lipoprotein cholesterol, high-sensitivity C-reactive protein, and lipoprotein(a) each to future cardiovascular events and death after acute coronary syndrome on high-intensity statin therapy: an analysis of the placebo arm of ODYSSEY OUTCOMES. Circulation 2025;151:1047–50. 10.1161/CIRCULATIONAHA.124.071269 40193536

[7] Vrints C, Andreotti F, Koskinas KC, Rossello X, Adamo M, Ainslie J, et al 2024 ESC guidelines for the management of chronic coronary syndromes. Eur Heart J 2024;45:3415–537. 10.1093/eurheartj/ehae177 39210710

[8] Kleemann R, Verschuren L, de Rooij BJ, Lindeman J, de Maat MM, Szalai AJ, et al Evidence for anti-inflammatory activity of statins and PPARalpha activators in human C-reactive protein transgenic mice in vivo and in cultured human hepatocytes in vitro. Blood 2004;103:4188–94. 10.1182/blood-2003-11-3791 14976045

[9] Voleti B, Agrawal A. Statins and nitric oxide reduce C-reactive protein production while inflammatory conditions persist. Mol Immunol 2006;43:891–6. 10.1016/j.molimm.2005.06.045 PMC3818092 16054696

[10] Schwartz GG, Steg PG, Szarek M, Bhatt DL, Bittner VA, Diaz R, et al Alirocumab and cardiovascular outcomes after acute coronary syndrome. N Engl J Med 2018;379:2097–107. 10.1056/NEJMoa1801174 30403574

[11] Trion A, de Maat MP, Jukema JW, van der Laarse A, Maas MC, Offerman EH, et al No effect of C-reactive protein on early atherosclerosis development in apolipoprotein E*3-leiden/human C-reactive protein transgenic mice. Arterioscler Thromb Vasc Biol 2005;25:1635–40. 10.1161/01.ATV.0000171992.36710.1e 15920036

[12] Rizo-Téllez SA, Sekheri M, Filep JG. C-reactive protein: a target for therapy to reduce inflammation. Front Immunol 2023;14:1237729. 10.3389/fimmu.2023.1237729 PMC10410079 37564640

[13] Akita K, Isoda K, Sato-Okabayashi Y, Kadoguchi T, Kitamura K, Ohtomo F, et al An interleukin-6 receptor antibody suppresses atherosclerosis in atherogenic mice. Front Cardiovasc Med 2017;4:84. 10.3389/fcvm.2017.00084 PMC5743912 29312959

[14] Interleukin-6 Receptor Mendelian Randomisation Analysis (IL6R MR) Consortium; Swerdlow DI, Holmes MV, Kuchenbaecker KB, Engmann JE, Shah T, et al The interleukin-6 receptor as a target for prevention of coronary heart disease: a Mendelian randomisation analysis. Lancet 2012;379:1214–24. 10.1016/S0140-6736(12)60110-X PMC3316968 22421340

[15] IL6R Genetics Consortium Emerging Risk Factors Collaboration; Sarwar N, Butterworth AS, Freitag DF, Gregson J, Willeit P, et al Interleukin-6 receptor pathways in coronary heart disease: a collaborative meta-analysis of 82 studies. Lancet 2012;379:1205–13. 10.1016/S0140-6736(11)61931-4 PMC3316940 22421339

[16] Zenti MG, Altomari A, Lupo MG, Botta M, Bonora E, Corsini A, et al From lipoprotein apheresis to proprotein convertase subtilisin/kexin type 9 inhibitors: impact on low-density lipoprotein cholesterol and C-reactive protein levels in cardiovascular disease patients. Eur J Prev Cardiol 2018;25:1843–51. 10.1177/2047487318792626 30058841

[17] Navarese EP, Podhajski P, Gurbel PA, Grzelakowska K, Ruscio E, Tantry U, et al PCSK9 inhibition during the inflammatory stage of SARS-CoV-2 infection. J Am Coll Cardiol 2023;81:224–34. 10.1016/j.jacc.2022.10.030 PMC9842071 36653090

[18] Ridker PM, Libby P, MacFadyen JG, Thuren T, Ballantyne C, Fonseca F, et al Modulation of the interleukin-6 signalling pathway and incidence rates of atherosclerotic events and all-cause mortality: analyses from the Canakinumab Anti-Inflammatory Thrombosis Outcomes Study (CANTOS). Eur Heart J 2018;39:3499–507. 10.1093/eurheartj/ehy310 30165610

[19] Kleveland O, Ueland T, Kunszt G, Bratlie M, Yndestad A, Broch K, et al Interleukin-6 receptor inhibition with tocilizumab induces a selective and substantial increase in plasma IP-10 and MIP-1β in non-ST-elevation myocardial infarction. Int J Cardiol 2018;271:1–7. 10.1016/j.ijcard.2018.04.136 29961572

[20] Broch K, Anstensrud AK, Woxholt S, Sharma K, Tøllefsen IM, Bendz B, et al Randomized trial of interleukin-6 receptor inhibition in patients with acute ST-segment elevation myocardial infarction. J Am Coll Cardiol 2021;77:1845–55. 10.1016/j.jacc.2021.02.049 33858620

[21] Ridker PM, Devalaraja M, Baeres FMM, Engelmann MDM, Hovingh GK, Ivkovic M, et al IL-6 inhibition with ziltivekimab in patients at high atherosclerotic risk (RESCUE): a double-blind, randomised, placebo-controlled, phase 2 trial. Lancet 2021;397:2060–9. 10.1016/S0140-6736(21)00520-1 34015342

[22] Imbesi A, Greco A, Capodanno D. Anti-inflammatory strategies for patients with atherosclerotic coronary disease. Expert Rev Cardiovasc Ther 2025;23:703–21. 10.1080/14779072.2025.2564137 40968010

