# Supplementary material for: Interleukin-6 and C-reactive protein prognostic value following acute coronary syndrome
Source: Eur Heart J. 2026 May 26;47(40):6008–17. doi: 10.1093/eurheartj/ehag317 (PMC13696072; doi:10.1093/eurheartj/ehag317)
Supplement: Supplementary file 1 [file ehag317_supplementary_data.docx]

**Supplementary Appendix**

**Interleukin-6 and C-reactive protein prognostic value following acute coronary syndrome**

J. Wouter Jukema et al.

Contents

[Appendix Table 1 Patient characteristics and markers of inflammation at baseline and at month 4 of assigned treatment	2](#_Toc220401712)

[Appendix Table 2 Concordance* of baseline hsCRP and IL-6 as predictors of MACE or death	4](#_Toc220401713)

[Appendix Table 3 Change in log(hsCRP) and log(IL-6) baseline to month 4 versus MACE^a^ and death	5](#_Toc220401714)

# Appendix Table 1 Patient characteristics and markers of inflammation at baseline and at month 4 of assigned treatment

| Variable | Alirocumab  (*n* = 5911) | Placebo  (*n* = 5906) |
| --- | --- | --- |
| Age, years | 58 (52, 65) | 58 (52, 65) |
| Female sex | 1409 (23.8) | 1422 (24.1) |
| Region of enrolment |  |  |
| Central and Eastern Europe | 1140 (19.3) | 1132 (19.2) |
| Western Europe | 1396 (23.6) | 1403 (23.8) |
| Canada or United States | 1391 (23.5) | 1391 (23.6) |
| Asia | 686 (11.6) | 685 (11.6) |
| Rest of World | 512 (8.7) | 517 (8.8) |
| Hypertension | 3766 (63.7) | 3689 (62.5) |
| HbA1c, % | 5.8 (5.5, 6.3) | 5.8 (5.5, 6.4) |
| eGFR, mL/min/1.73m^2^ | 77.9 (67.2, 90.1) | 78.2 (67.4, 90.5) |
| Diabetes | 1724 (29.2) | 1762 (29.8) |
| Current smoking | 1420 (24.0) | 1429 (24.2) |
| High-intensity statin treatment at randomization | 5234 (88.5) | 5285 (89.5) |
| Index acute coronary syndrome | *n* = 5902 | *n* = 5897 |
| Non–ST-segment elevation myocardial infarction | 2819 (47.7) | 2835 (48.0) |
| ST-segment elevation myocardial infarction | 2147 (36.3) | 2107 (35.7) |
| Unstable angina | 936 (15.8) | 955 (16.2) |
| Time from index ACS to randomization, months | 2.7 (1.7, 4.5) | 2.7 (1.8, 4.5) |
| Revascularization for index ACS | 4300 (72.7) | 4363 (73.9) |
| Baseline markers of inflammation |  |  |
| hsCRP, mg/L | 1.50 (0.73, 3.45) | 1.58 (0.74, 3.71) |
| Interleukin-6 pg/mL | 4.97 (3.79, 6.81) | 5.03 (3.84, 6.97) |
| Baseline lipids and lipoproteins |  |  |
| LDL-C, mg/dL | 86 (73, 104) | 86 (73, 105) |
| Triglycerides, mg/dL | 129 (95, 182) | 131 (95, 186) |
| HDL-C, mg/dL | 42 (36, 50) | 42 (36, 50) |
| Lipoprotein(a), nmol/L | 44.5 (13.4, 150.8) | 45.5 (13.1, 157.5) |
| Month 4 hsCRP values | (n = 5478) | (n = 5455) |
| Month 4, mg/L | 1.42 (0.70, 3.17) | 1.40 (0.69, 3.19) |
| Absolute change from baseline | –0.04 (-0.84, 0.59) | –0.08 (–0.94, 0.53) |
| Per cent change from baseline | –4.0 (–41.5, 54.8) | –8.1 (–45.2, 51.6) |
| Month 4 interleukin-6 values | (n = 5478) | (n = 5455) |
| Month 4, mg/L | 4.74 (3.61, 6.46) | 4.80 (3.60, 6.67) |
| Absolute change from baseline | –0.25 (–1.79, 1.19) | –0.22 (–1.74, 1.15) |
| Per cent change from baseline | –4.9 (–30.5, 27.5) | –4.5 (–30.5, 26.6) |
| Month 4 LDL-C values | (n = 5478) | (n = 5455) |
| Month 4, mg/dL | 30 (20, 46) | 88 (73, 108) |
| Absolute change from baseline | –55 (–71, –39) | 1 (–12, 15) |
| Per cent change from baseline | –65.3 (–76.1, –49.4) | 1.1 (–13.0, 18.0) |

Numbers are n (%) or median (inter-quartile range).

ACS, acute coronary syndrome; eGFR, estimated glomerular filtration rate; HbA1c, glycated haemoglobin; hsCRP, high-sensitivity C-reactive protein; HDL-C, high-density lipoprotein cholesterol; LDL-C, low-density lipoprotein cholesterol.

# Appendix Table 2 Concordance* of baseline hsCRP and IL-6 as predictors of MACE or death

| Model number and predictors | Concordance (95% CI) | Difference vs. model 1 (95% CI) | Difference vs. model 1 *P*-value | Difference vs. model 2 (95% CI) | Difference vs. model 2 *P*-value |
| --- | --- | --- | --- | --- | --- |
| Baseline hsCRP and IL-6 as predictors of MACE |  |  |  |  |  |
| 1. Treatment assignment, age, sex, diabetes, baseline LDL-C, and time from index ACS to randomization + Log(hsCRP) spline | 50.0%  (46.7%, 53.3%) | -- | -- | -- | -- |
| 2. Treatment assignment, age, sex, diabetes, baseline LDL-C, and time from index ACS to randomization + Log(IL-6) spline | 49.2%  (45.8%, 52.6%) | -- | -- | -- | -- |
| 3. Treatment assignment, age, sex, diabetes, baseline LDL-C, and time from index ACS to randomization + Log(hsCRP) spline + Log(IL-6) spline | 62.5%  (59.3%, 65.7%) | 12.5%  (6.8%, 18.2%) | <0.0001 | 13.3%  (7.5%, 19.1%) | <0.0001 |
| Baseline hsCRP and IL-6 as predictors of death |  |  |  |  |  |
| 1. Treatment assignment, age, sex, diabetes, baseline LDL-C, and time from index ACS to randomization + Log(hsCRP) spline | 62.6%  (59.0%, 66.2%) | -- | -- | -- | -- |
| 2. Treatment assignment, age, sex, diabetes, baseline LDL-C, and time from index ACS to randomization + Log(IL-6) spline | 62.5%  (58.9%, 66.1% | -- | -- | -- | -- |
| 3. Treatment assignment, age, sex, diabetes, baseline LDL-C, and time from index ACS to randomization + Log(hsCRP) spline + Log(IL-6) spline | 70.2%  (65.9%, 74.6%) | 7.6%  (3.3%, 12.0%) | 0.0006 | 7.7%  (3.5%, 11.9%) | 0.0003 |

ACS, acute coronary syndrome; CI, confidence interval; hsCRP, high-sensitivity C-reactive protein; IL-6, interleukin-6; LDL-C, low-density lipoprotein cholesterol; MACE, major adverse cardiovascular events.

^a^ The proportion of pairs of participants for whom observed and predicted MACE outcomes agree (are concordant) among all possible pairs in which one participant has a MACE and the other participant does not.

^b^ The proportion of pairs of participants for whom observed and predicted all-cause death outcomes agree (are concordant) among all possible pairs in which one participant dies and the other participant does not.

# Appendix Table 3 Change in log(hsCRP) and log(IL-6) baseline to month 4 versus MACE^a^ and death

| Outcome | Change Variable | HR (95% CI) for  1 SD increase | *P*-value | Treatment *P*_interaction_ |
| --- | --- | --- | --- | --- |
| MACE (10 757 patients, 980 events) | log(hsCRP) | 1.201 (1.116, 1.292) | <0.0001 | 0.24 |
|  | log(IL-6) | 1.100 (1.017, 1.189) | 0.0169 | 0.68 |
| Death (10 933 patients, 361 events) | log(hsCRP) | 1.197 (1.068, 1.341) | 0.0020 | 0.24 |
|  | log(IL-6) | 1.219 (1.094, 1.359) | 0.0003 | 0.29 |

CI, confidence interval; hsCRP, high-sensitivity C-reactive protein; HDL-C, high-density lipoprotein cholesterol; IL-6, interleukin-6; LDL-C, low-density lipoprotein cholesterol; MACE, major adverse cardiovascular events; SD, standard deviation.

Results reflect a single model for each endpoint (i.e. both change variables in the model) with adjustment for baseline log(hsCRP), baseline log(IL-6), treatment assignment, age, sex, diabetes, baseline LDL-C and time to index ACS.

1 SD increase = 1.00 for hsCRP, 0.66 for IL-6.

^a^MACE results only include events after month 4 assessments of hsCRP and IL-6.
